# Forming Characteristics of Tailor Rolled Blank of Aluminum Alloy during Three-Point Bending

**DOI:** 10.3390/ma17030591

**Published:** 2024-01-25

**Authors:** Ying Zhi, Yue Feng, Dong Wang, Xianlei Hu, Tao Sun, Xianghua Liu

**Affiliations:** 1 State Key Laboratory of Rolling and Automation, Northeastern University, Shenyang 110819, China; 2 School of Material Science and Engineering, Northeastern University, Shenyang 110819, China

**Keywords:** tailor rolled blank, aluminum alloy, spring-back characteristic, three-point bending, metal flow

## Abstract

This paper presents an investigation on the forming characteristics of the tailor rolled blank of an aluminum alloy (Al-TRB) during three-point bending at room temperature through experiments and finite element simulations. The strain distribution, spring-back characteristics, and metal flow law of 6000 series Al-TRB during three-point bending are explored. The prepared Al-TRB has good bending properties, and no surface cracks appear in the bending region of the Al-TRB when bent to 180°. Surface roughening occurs on the outside of the bending region. Since the strain in the thick zone is greater than that in the thin zone, the surface roughening in the thick zone is more obvious than that in the thin zone. The spring-back angle in the thin zone is higher than that in the thick zone after three-point bending, and the overall spring-back angle of Al-TRB becomes larger with an increasing bending angle. When the transition zone of Al-TRB is centered and the length of the transition zone is certain, as the length of the equal-thickness zone increases, the spring-back angle of the thin zone is larger, while the spring-back angle of the thick zone is smaller. Under the premise of a certain total length of Al-TRB and the length of the transition zone, the larger the length proportion of the thin zone, the larger the overall spring-back angle of Al-TRB, and the larger the length proportion of the thick zone, the smaller the overall spring-back angle of Al-TRB. In addition, a slight metal flow phenomenon exists during three-point bending, which shows that the metal in the bending region will flow to the thick zone, and the metal at the edge will flow to the thin zone. At the same time, there are localized thickening and thinning phenomena in Al-TRB. This study is helpful because it provides theoretical guidance for designing molds for the actual production of Al-TRB parts for automotives.

## 1. Introduction

Variable-thickness blanks are used in automobile, shipbuilding, bridgebuilding, and other industries, which have the advantages of saving material and weight, lowering costs, and improving efficiency. Variable-thickness blanks generally include tailor welded blanks (TWBs) and tailor rolled blanks (TRBs) [1]. TRBs produced by variable gauge rolling (VGR) has a thin zone, thick zone, and transition zone. Compared with a TWB, a TRB has no weld, good quality, a low production cost, and high efficiency, and the length and shape of the transition zone can be designed according to the stress conditions of the stamped parts in service. TRBs have been receiving a lot of attention because of their more obvious material saving and weight reduction effects [2,3,4]. TRBs have been applied in the manufacture of automotive lightweight parts at home and abroad. Many automotive lightweight parts can be made from TRBs, including B-pillars, crash beams, front longitudinal beams, etc. Currently, TRBs of low-alloy steel and TRBs of hot forming steel are more commonly used [5,6,7].

Aluminum alloys, as lightweight materials used in new energy vehicles, can effectively reduce the weight of the body, reduce energy consumption, and improve power [8,9,10,11]. If VGR technology and aluminum alloys can be combined, they will advance the further development of automotive lightweighting. Ideal tailor rolled blanks of aluminum alloy (Al-TRB) parts have a weight reduction of at least 20–30% compared with equal-thickness parts; at the same time, the material utilization rate will exceed 60%, which can be applied in automotive chassis components, cross beams, door parts, seat parts, and body parts [12]. Therefore, the research and application of Al-TRB for automobiles have revealed that it is a characteristic new technology for energy and material saving, and it has a good prospect for development.

The 6000 series aluminum alloys have excellent comprehensive performance with medium strength, good welding performance, corrosion resistance, and surface quality, and there is a growing tendency at home and abroad to develop automobile body coverings and structural parts based on 6000 series aluminum alloys [13,14,15,16]. At present, the industrial production of Al-TRB has not yet been realized. The Mubea company has conducted a pilot study of 6000 series Al-TRB on its production line to explore the feasibility of the industrial production of Al-TRB [12]. Little research has been conducted on the organization and properties of Al-TRB, especially the forming characteristics. There is a lack of systematic and in-depth research on the press-forming properties of 6000 series Al-TRB obtained through VRG and heat treatment.

The spring-back characteristic, as the most common problem in the cold-press forming process, has a great impact on the dimensional accuracy and productivity of parts [17,18,19]. The modulus of elasticity of an aluminum alloy is about one-third that of steel, and the spring-back of an aluminum alloy blank during cold-press forming is much greater than that of steel blank [20]. Therefore, Al-TRB after cold-press forming also has the characteristic of a large spring-back, so the difficulty of controlling the dimensional accuracy after forming increases. At the same time, the complexity of the dimensional characteristics and the differentiation of mechanical properties of TRB further exacerbate the difficulty of dimensional control after cold-press forming [21,22,23]. Metal flow in the TRB during the cold stamping process can lead to offsets in the transition zone as well as localized thinning and thickening, all of which affect the accuracy of dimensional control of the part [24]. So, it is necessary to study the spring-back characteristics, strain distribution, and metal flow law of Al-TRB.

In this study, three-point bending experiments at room temperature and finite element simulations were carried out on 6000 series Al-TRB obtained by VGR and heat treatment. The strain distribution, spring-back characteristics, and metal flow law of Al-TRB during three-point bending were explored, which will provide theoretical guidance for designing molds for the actual production of Al-TRB parts and promote the application of Al-TRB in automotive lightweighting.

## 2. Experimental Materials and Research Methods

### 2.1. VGR and Heat Treatment Processes of Aluminum Alloy

The experimental raw material is a 6000 series aluminum alloy sheet with a length of 300 mm, a width of 300 mm, and an equal thickness of 3.0mm (T4P), and its chemical composition is shown in Table 1.

**Figure 1:**
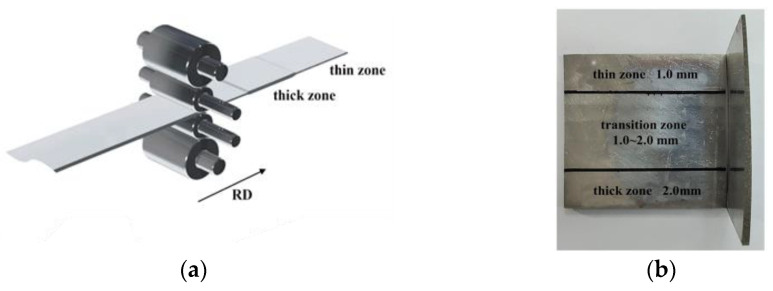
Schematic diagram of VGR and Al-TRB: (**a**) diagram of VGR; (**b**) Al-TRB cut into square objects.

VGR experiment was carried out on a four-high cold rolling mill (RAL, Shenyang, China) to roll the raw material into Al-TRB with the thicknesses of 1.0 mm and 2.0 mm in the thin and thick zones, respectively. The ratio of variable gauge of TRB was 1:2, and the slope of the transition zone was 1:30. The schematic diagram of VGR and the dimensions of the obtained Al-TRB are shown in Figure 1.

The heat treatment experiment was conducted on Al-TRB obtained by VGR. Firstly, Al-TRB was subjected to solution treatment with a solution temperature of 540℃ and a solution time of 10 min. Then, the solution-treated Al-TRB was subjected to pre-aging treatment with a pre-aging temperature of 120 °C and a pre-aging time of 10 min, and Al-TRB with the T4P state was obtained. Al-TRB obtained using this process has small differences in mechanical properties between thin and thick zones, high bake-hardening capacity, and excellent overall mechanical properties. Tensile tests were conducted on thin and thick zones of Al-TRB using CMT5105-SANC tensile machine (MTS, Shenzhen, China). 

To assign values to the mechanical properties of the transition zone in FEM simulation studies, the partition discretization was widely used to approximate the mechanical properties of transition zone of TRB. Four equal-thickness blanks with thicknesses of 1.2 mm, 1.4 mm, 1.6 mm, and 1.8 mm were prepared by cold rolling and then later heat-treated with the same process as Al-TRB. Finally, tensile experiments were conducted to obtain the mechanical properties of different equal-thickness zones. 

### 2.2. Experiment and Test Method of Spring-Back on Three-Point Bending of Al-TRB

Three-point bending experiments were used to study the spring-back properties of Al-TRB. Due to the limitations of the size of the experimental equipment, the dimensions of the Al-TRB used for experimental research refer to the international standard VDA238-100 [25], and the size of the bending part is 60 mm × 60 mm (RD × TD). The thicknesses of the thin zone and the thick zone are 1.0 mm and 2.0 mm, respectively, and both lengths are 15 mm. The transition zone is centered and linear with a length of 30 mm.

**Figure 2:**
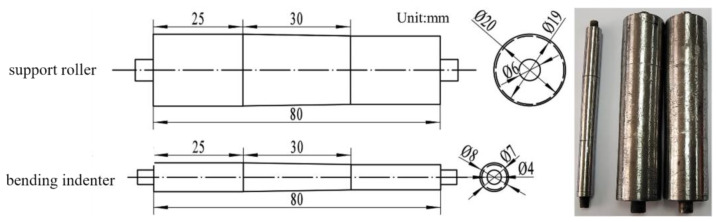
Dimensions and physical objects of the support roller and bending indenter.

Due to the thickness variation in the blank itself, a set of three-point bending molds with variable thickness needs to be designed for spring-back studies in order to achieve a perfect fit with the blank. The dimensions and the physical objects of the two designed support rolls with identical dimensions and bending indenter are shown in Figure 2.

**Figure 3:**
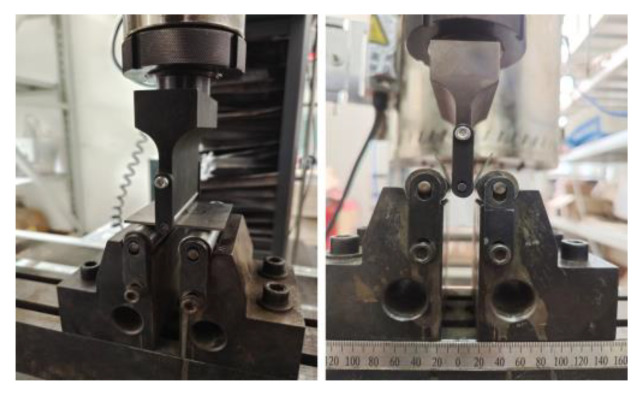
CMT 5105-SANS tensile testing machine for three-point bending test.

CMT5105-SANC with a span of 34 mm was selected as the experimental equipment for three-point bending. When Al-TRB bending parts are placed, the bending indenter should be placed across the thin zone, transition zone, and thick zone. The thin zone was placed on the larger side of the bending indenter roll diameter, the transition zone of Al-TRB was aligned with the transition zone of the bending indenter, and the thick zone was placed on the smaller side of the bending indenter roll diameter. The pressing speed of bending indenter was 2 mm/min, and the experimental method is shown in Figure 3.

**Figure 4:**
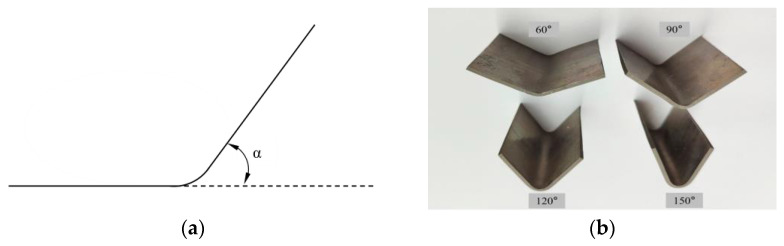
Diagram of bending angle of Al-TRB: (**a**) representation of bending angle; (**b**) Al-TRB with different bending angles.

**Figure 5:**
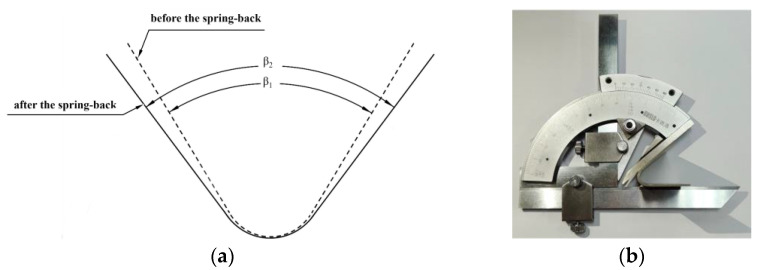
Representation and measurement method of angle: (**a**) representation of spring-back angle; (**b**) measurement method for angle.

In order to investigate the spring-back of different bending degrees and the cracking of the bending region, five bending angles of 60°, 90°, 120°, 150°, and 180° were set, such as the α angle in Figure 4a. The bending parts with a 180° bending angle were used for crimping performance tests, and four bending parts with four bending angles of 30°, 60°, 90°, and 120° were used for spring-back studies, as shown in Figure 4b. The angles made by the two sides of the sheet before and after spring-back are denoted by β_1_ and β_2_, respectively, and the final spring-back angle, θ, is equal to β_2_–β_1_, as shown in Figure 5a. The angle formed by the sides of the sheet in the thin and thick zones after spring-back were measured with a protractor, as shown in Figure 5b. The three-point bending experiment was repeated three times for each bending angle, and the final average of the three spring-back results was taken as θ. 

## 3. Experimental Results and Analysis

### 3.1. Analysis of Spring-Back Results

The spring-back angles of Al-TRB after three-point bending are shown in Table 2. It can be seen that the spring-back angle in the thin zone of Al-TRB is larger than that in the thick zone, and the spring-back phenomenon is more obvious. The maximum difference in the spring-back angle between the thin and thick zones is 2.5° when the bending angle is 180°. With the increase in the bending angle, the spring-back angles of the thin and thick zones of the Al-TRB bending parts are gradually increased. The size of the spring-back angle in the thin and thick zones is greatly related to the differences in their mechanical properties and the work hardening rate of the deformation process.

### 3.2. Bending Performance Test

**Figure 6:**
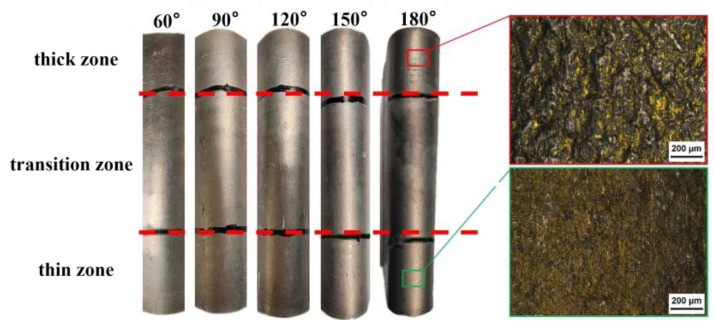
Surface micrographs on the outside of the bending region of Al-TRB.

The outer and inner panels of automobiles are connected by crimping, which requires the panels to have certain bending properties. An aluminum alloy sheet will exhibit an “orange peel” effect of surface coarsening in the deformation area. The SAE (Society of Automotive Engineers) standard [26] shows that the surface roughening phenomena can effectively be suppressed by conducting proper thermo-mechanical processing (TMP) on the material. The surface roughening is improved by painting after press forming, so the surface roughening has almost no effect on the final paint appearance of the sheet after painting and baking [26]. Figure 6 shows the surface morphology of the outside of the bending region of Al-TRB with different bending angles, as well as the metallographic photos of the thin and thick regions at a magnification of 50 times when the bending angle is 180°. It can be seen that the surface roughening phenomenon in the thick zone is more obvious than that in the thin zone, and the surface roughening phenomenon on the outside of the bending region becomes more obvious as the bending angle increases. However, no surface cracks are observed in the Al-TRB when the bending angle is 180°, which indicates that the prepared Al-TRB in the T4P state has good bending properties.

## 4. Finite Element Simulation of Forming Characteristics during Three-Point Bending

Due to the limitation of the experimental equipment, the size of the experimental Al-TRB is small, and the length of the Al-TRB in the actual application may be much larger than 60 mm. Therefore, it is necessary to simulate and analyze the three-point bending process of Al-TRB by using the ABAQUS software (Version 2022) to establish a finite element model. The accuracy of the simulation results is verified by comparing the experimental results with the simulation results. The strain distribution, spring-back characteristics, and metal flow law of the Al-TRB during three-point bending are further simulated and analyzed.

### 4.1. Finite Element Modeling for Three-Point Bending and Forming

In the finite element analysis, the dimensions of the bending indenter and support roll are kept consistent with those of the experiment, and the dimensions of the Al-TRB vary depending on the study content. In order to verify the reliability of the simulation results, the dimensions of the Al-TRB will be consistent with the dimensions of the experimental Al-TRB bending part, and the size of the bending part will still be 60 mm × 60 mm (RD × TD). The thickness of the thin zone and the thick zone are 1.0 mm and 2.0 mm, respectively, and both lengths are 15 mm. The transition zone is centered and linear with a length of 30 mm. Depending on the content of the analysis, the length of the TRB and the location of the transition zone may change later in the text. In this paper, four different sizes of Al-TRB are used for the subsequent simulation studies with dimensional parameters, as shown in Table 3.

**Figure 7:**
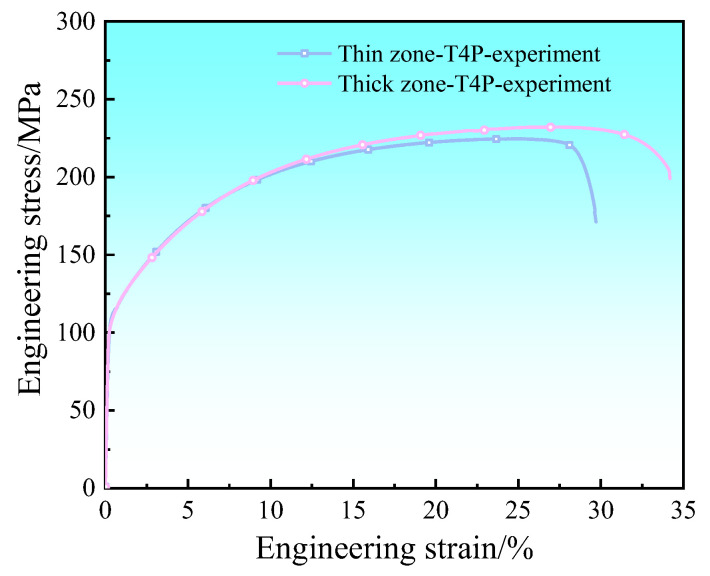
Engineering stress–strain curves in the thin and thick zones of the T4P state Al-TRB.

**Figure 8:**
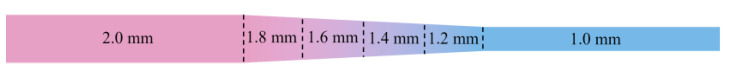
Discrete treatment of mechanical properties in the transition zone.

The engineering stress–strain curves in the thin and thick zones of the T4P state Al-TRB measured using a tensile experiment are shown in Figure 7. In the thin zone of the Al-TRB, the measured yield strength is 100 MPa, the tensile strength is 224 MPa, and the elongation at break is 29.7%. In the thick zone of the Al-TRB, the measured yield strength is 99 MPa, the tensile strength is 232 MPa, and the elongation at break is 35.1%. The mechanical properties in the transition zone of the Al-TRB in the T4P state undergo a linear transition from the thin zone to the thick zone. Therefore, under the premise of ensuring the simulation accuracy, the transition zone is discretized into equal-thicknesses parts with four thicknesses of 1.2 mm, 1.4 mm, 1.6 mm, and 1.8 mm, as shown in Figure 8, and their mechanical properties are obtained from tensile tests. The mechanical properties at any thickness of the Al-TRB transition zone were obtained by linear interpolation so as to establish the material property model of the Al-TRB transition zone.

**Figure 9:**
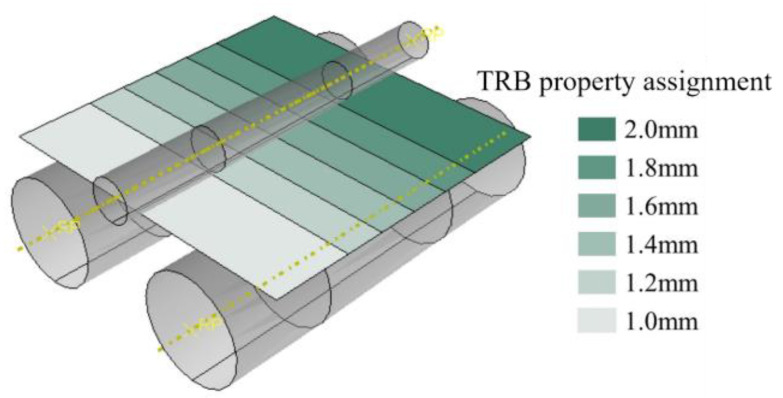
Assembly diagram of the three-point bending model.

Shell units were used for sheet modeling, which can prevent the shear self-locking phenomenon of solid cells during bending deformation. An all-quadrilateral grid with a S4RT cell type and a cell size of 0.4 mm was used with seven integration points in the thickness direction. The bending indenter and the lower support roller are discrete rigid bodies with variable thickness designs, and these rigid body parts have no deformation during three-point bending process. The assembly diagram of the three-point bending model is shown in Figure 9.

**Figure 10:**
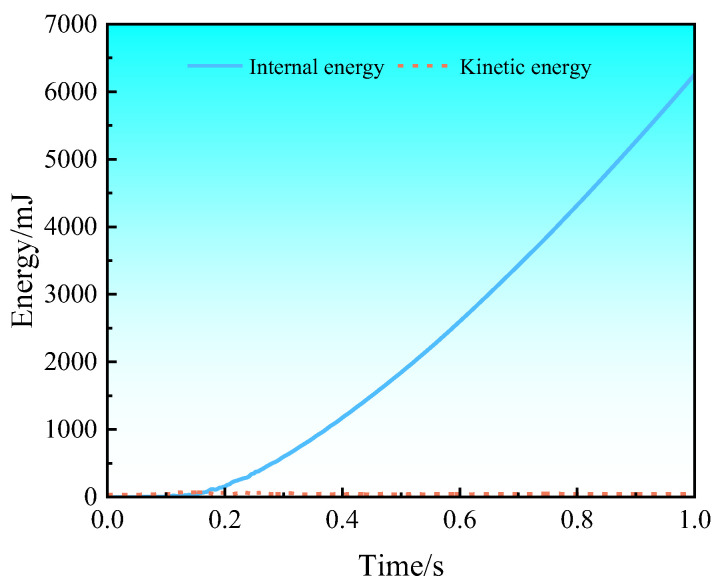
Changes in kinetic energy and internal energy calculated during simulation.

The three-point bending process was completed in one analysis step using the dynamic display algorithm. In order to speed up the calculation, a mass amplification factor was used, which has the effect of amplifying the calculation time of a single step close to the set target time of 1 × 10^−5^ s. The changes in kinetic energy and internal energy calculated using the ABAQUS software during three-point bending are shown in Figure 10. It can be seen that the ratio of kinetic energy to internal energy is much less than 5%, indicating that mass amplification has no significant effect on the calculated results of three-point bending, and the calculated results are real and reliable.

### 4.2. Strain States of Al-TRB at Different Bending Angles

**Figure 11:**
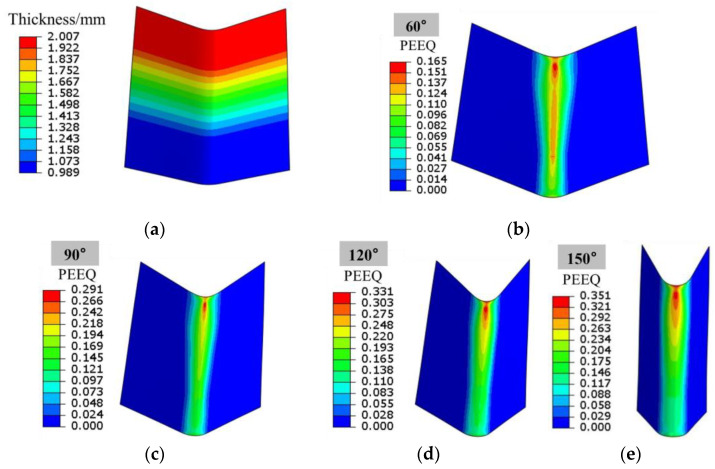
Equivalent strain distribution of Al-TRB after bending: (**a**) Al-TRB thickness distribution; (**b**) bending angle of 60°; (**c**) bending angle of 90°; (**d**) bending angle of 120°; (**e**) bending angle of 150°.

Studying the strain state of Al-TRB after bending into different angles helps to analyze the deformation, surface roughening, crimping performance, and failure cracking of different parts during the bending process. The size of the Al-TRB is set at 60 mm × 60 mm (RD × TD), and the transition zone is located in the middle of the TRB, i.e., Al-TRB-Ⅰ. The bending indenter is pressed down by 8.4 mm, 11.9 mm, 15.8 mm, and 22.35 mm, at which time the bending angles are 60°, 90°, 120°, and 150°, respectively. Figure 11 shows the equivalent strain distribution of the Al-TRB when bending into different angles, and the strain in the bending region of the Al-TRB increases with the increase in the bending degree. The strain concentration in the bending process is mainly distributed in the thick zone, and the strain in the thick zone is larger than that in the thin region, which is the reason why surface roughening is more pronounced in thick zones than in thin zones. When the bending angle reaches 90°, the strain extreme value of 0.291 appears in the bending region of the thick zone. When the bending angle reaches 150°, the extreme value reaches 0.351.

### 4.3. Analysis of Spring-Back Properties on Three-Point Bending of Al-TRB

The spring-back behavior of the Al-TRB after three-point bending is related to many factors, such as the bending degree of the TRB, the dimensions of the TRB, and the downward speed of the bending indenter. In this section, only the effects of the degree of bending of the TRB and the dimensional condition of the TRB on the spring-back behavior after bending are investigated.

#### 4.3.1. Spring-Back Behavior of Al-TRB at Different Bending Angles

**Figure 12:**
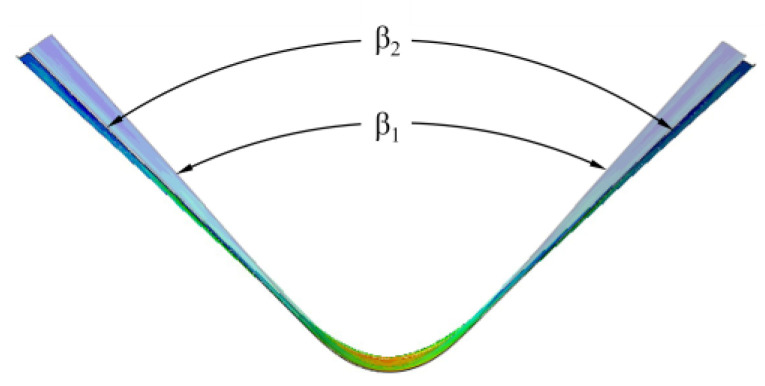
Cross-sectional shape of Al-TRB before and after spring-back (β_1_—before spring-back; β_2_—after spring-back).

Metal parts undergo a certain degree of spring-back behavior after cold-pressing, which directly affects the dimension accuracy of the final part. The Al-TRB after three-point bending is imported into the static analysis of finite element, and the bending part is allowed to stretch freely under certain boundary conditions so that the shape of the Al-TRB can be obtained after undergoing spring-back behavior, and thus, the angle of spring-back can be calculated. The Al-TRB needs to add a fully fixed constraint in the bottom of the bending region during spring-back, which can limit the movement of the blank and prevent the conversion of elastic potential energy into kinetic energy in the bending part during spring-back, so as to ensure the convergence and accuracy of the spring-back simulation. The cross-sectional shape of the simulated Al-TRB before and after spring-back is shown in Figure 12.

Table 4 shows the simulation results of the spring-back angles of the thin zone and thick zone of the Al-TRB after three-point bending. It can be seen that the spring-back angle of the thin zone is larger than that of the thick zone when the bending angle is the same, and the maximum difference in the spring-back angle between the thin and thick zones is 2.7° when the bending angle is 180°. The spring-back angle increases gradually with an increasing bending angle, which is consistent with the experimental results.

**Figure 13:**
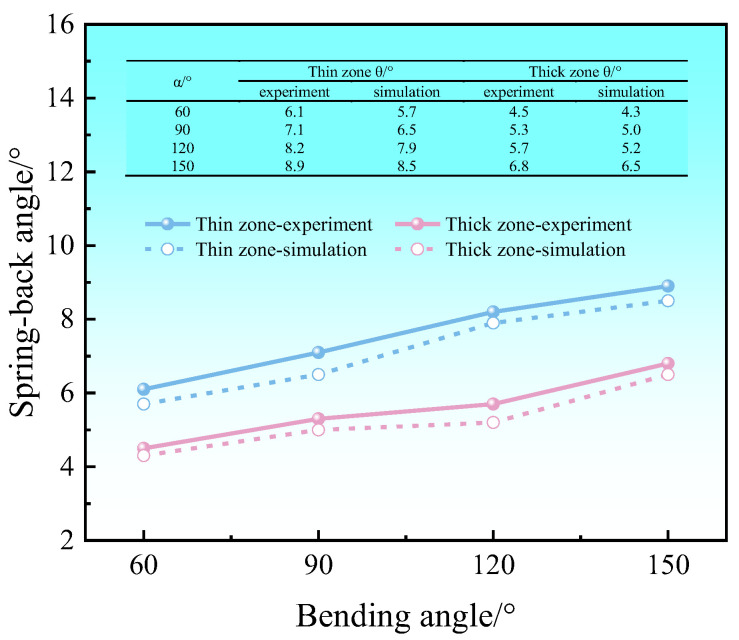
Comparison of experimental and simulated spring-back results for Al-TRB.

Figure 13 shows a comparison between the simulated and experimental results of spring-back. It can be seen that the simulated results of the spring-back angle of the Al-TRB are a little lower than the experimental results. The difference between the experimental and simulated results is small, and the maximum error of the spring-back angle is only 0.6°, which verifies the accuracy of the simulation results, and this finite element model can be used to continue the subsequent study on the effect of Al-TRB dimensions on spring-back.

#### 4.3.2. Influence of the Equal-Thickness Zone Length of Al-TRB on Spring-Back

**Figure 14:**
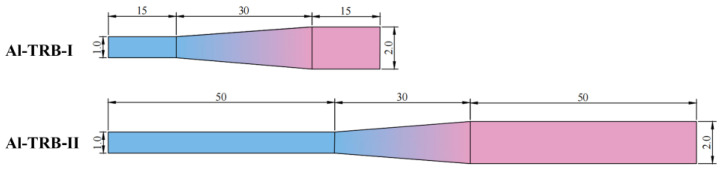
Dimensions of the two sizes of Al-TRB (mm).

The length of the actual automobile part is much larger than the length of the TRB set in the previous section. In order to investigate the influence of the length of the equal-thickness zone on the spring-back of the TRB, the length of the thin zone and the thick zone of the TRB is set to be extended from 15mm to 50 mm, and the length of the transition zone is still in the center with a length of 30 mm. The dimensions of the two sizes of Al-TRB studied in comparison, Al-TRB-I and Al-TRB-II, are shown in Figure 14 and Table 3.

**Figure 15:**
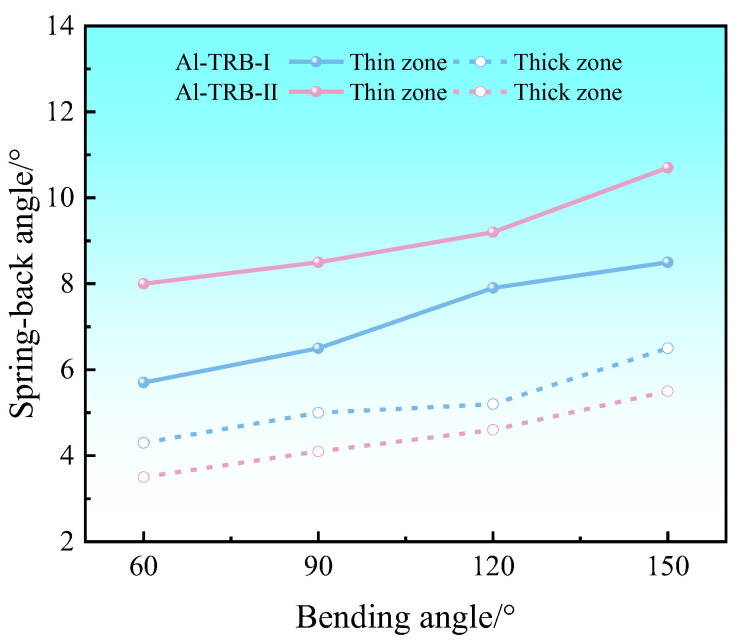
Effect of the length of the equal-thickness zone on the spring-back angles of Al-TRB.

The simulation results of the spring-back angle after the three-point bending of two sizes of Al-TRB are shown in Figure 15; the spring-back angle of the thin zone of the Al-TRB is significantly larger than that of the thick zone for both sizes. When the transition zone of the Al-TRB is centered and the length of the transition zone is certain, as the length of the equal-thickness zone increases, the spring-back angle of the thin zone is larger, while the spring-back angle of the thick zone is smaller. In fact, the length of many parts is very long, and the length of the required equal-thickness zone of the TRB is much larger than the length of the transition zone. The comparison of the bending spring-back properties of TRBs with different equal-thickness zone lengths shows that the spring-back angles of the thin and thick zones of some long TRB parts will be a little larger than the results shown in Figure 15.

#### 4.3.3. Influence of the Transition Zone Position of Al-TRB on the Spring-Back

**Figure 16:**
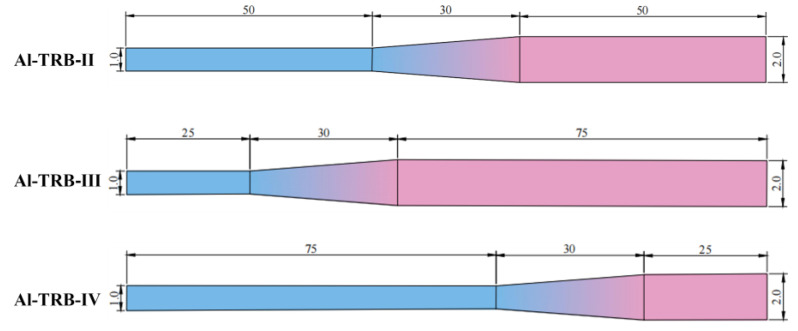
Dimensions of the three sizes of Al-TRB (mm).

The transition zone of the actual TRB component is not necessarily located in the middle of the TRB, so it is also necessary to study the effect of the location of the transition zone on the spring-back behavior of the TRB. The three sizes of Al-TRB mentioned earlier are used, provided that the total length is 130 mm in all cases and the length of the transition zone is 30 mm. The transition region of Al-TRB-II is centered. The lengths of the thin and thick zones of Al-TRB-III are 25 mm and 75 mm, and the transition zone is shifted to the thin zone compared to Al-TRB-II. The lengths of the thin and thick zones of Al-TRB-IV are changed to 75 mm and 25 mm, and the transition zone is shifted to the thick zone compared to Al-TRB-II. The dimensions of the three sizes of Al-TRB studied are shown in Figure 16 and Table 3.

**Figure 17:**
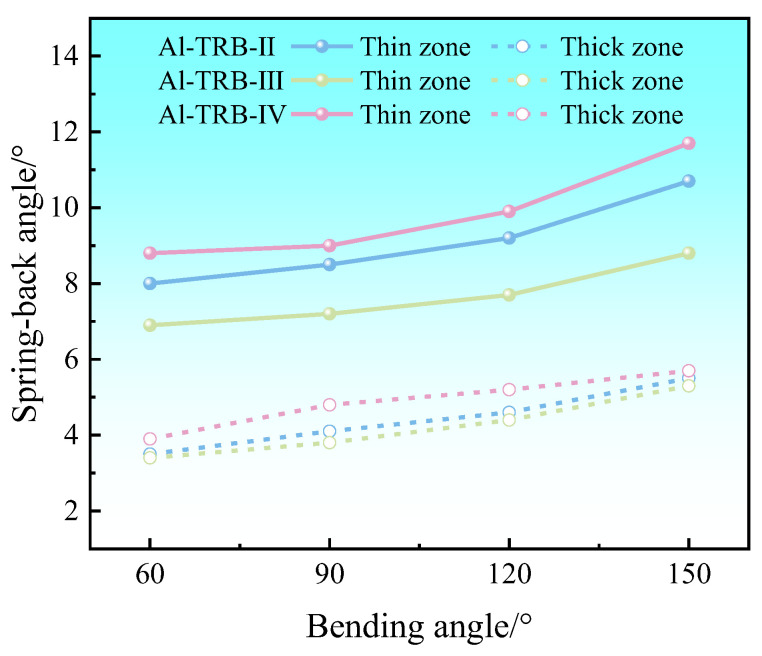
Spring-back angles after three-point bending of Al-TRB with different transition zone locations.

The effect of three transition zone positions on the spring-back angle of Al-TRB after three-point bending is shown in Figure 17. From the figure, it can be seen that the spring-back angles of the Al-TRB’s thin zone and thick zone decrease when the transition zone of the Al-TRB is biased toward the thin zone, the spring-back angle of the Al-TRB increases when the transition zone is biased toward the thick zone, and the spring-back angle of the thin zone is more obviously affected by the position of the transition zone than that of the thick zone.

The above results show that under the premise of a certain total length of Al-TRB and the length of the transition zone, changing the position of the transition zone results in a change in the ratio of the lengths of the thin and thick zones to the total length, which affects the overall spring-back of the Al-TRB. The larger the length proportion of the thin zone, the larger the overall spring-back angle of the Al-TRB, and the larger the length proportion of the thick zone, the smaller the overall spring-back angle of the Al-TRB. This is due to the existence of a neutral surface that does not deform in the thickness direction during the bending deformation of the Al-TRB and the existence of an elastic region that does not undergo plastic deformation in the vicinity of the neutral surface, which has a significant effect on the spring-back. Due to the obvious difference in the thicknesses of the thin and thick zones of the Al-TRB, the percentage of elastic deformation region in the thin zone is obviously larger than that in the thick zone, so the spring-back of the thin zone is obviously larger than that of the thick zone. Therefore, the larger the proportion of the length in the thin zone, the larger the overall spring-back angle of the Al-TRB bending part. However, the difference in the modulus of elasticity between the thin and thick zones is a secondary reason for the difference in spring-back between the thin and thick zones. The modulus of elasticity in the thin zone (69 GPa) is slightly smaller than the modulus of elasticity in the thick zone (73 GPa), so to some extent, the spring-back angle of the thin zone is slightly larger than that of the thick zone.

### 4.4. Metal Flow Law of Al-TRB during Three-Point Bending

The finite element method was used to investigate the metal flow law of the Al-TRB during three-point bending, and the offsets and thickness changes in the thin, thick, and transition zones of the Al-TRB after three-point bending were obtained, which can provide guidance for the control of the dimensional accuracy of the Al-TRB in the press-forming process.

#### 4.4.1. Offsets in Each Zone of Al-TRB after Three-Point Bending

**Figure 18:**
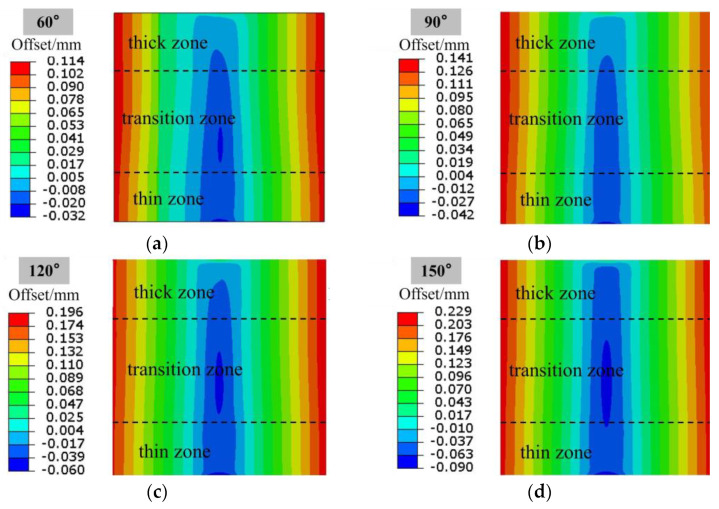
Position offset of each part of Al-TRB after three-point bending (the direction of thick zone pointing to thin zone is positive): (**a**) bending angle of 60°; (**b**) bending angle of 90°; (**c**) bending angle of 120°; and (**d**) bending angle of 150°.

It is also very important to study the metal flow law during the bending process of the Al-TRB, which is related to the offset of the thin zone, thick zone, and transition zone. The position offset of each part of the Al-TRB when bending into different angles is shown in Figure 18, and the direction of metal flow from the thick zone to the thin zone is set to positive. The three-point bending of Al-TRB shows the trend that the metal in the bending region flows to the thick zone, and the metal in the edge part flows to the thin zone. The offsets in the bending region and the edge part of the Al-TRB increase with the increase in the bending degree.

**Figure 19:**
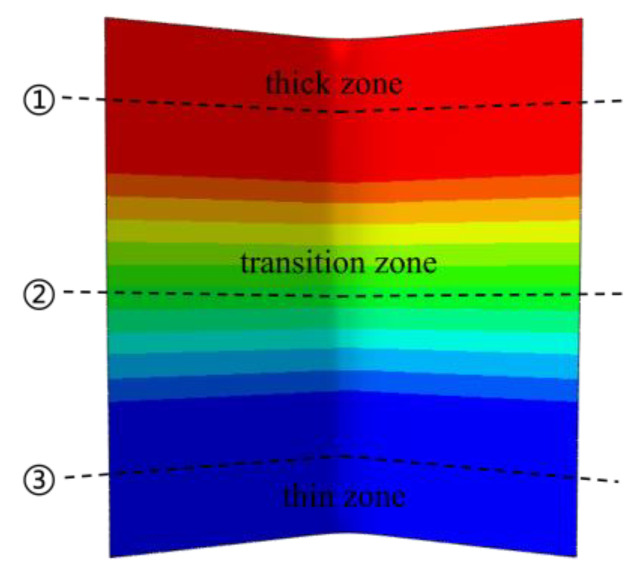
Diagram of the examination path of the offset in each thickness zone of Al-TRB.

**Figure 20:**
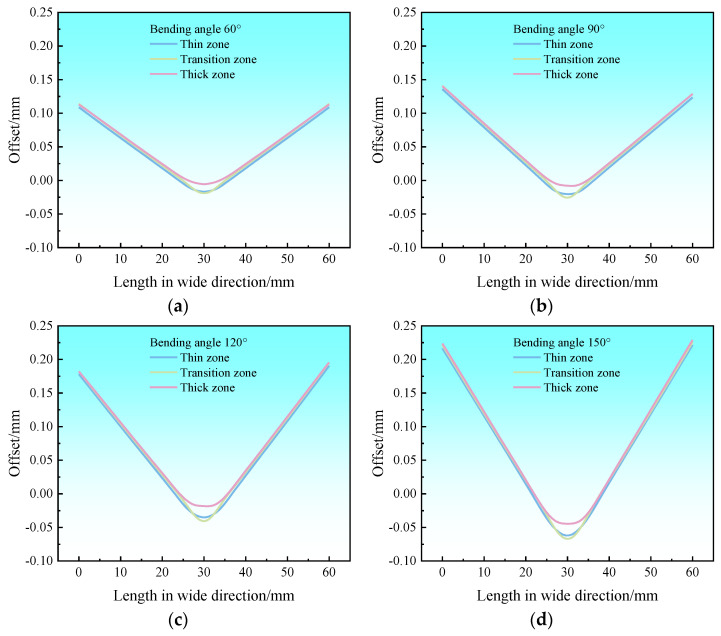
Variation in offset along the width direction in each thickness zone of Al-TRB for different bending angles: (**a**) bending angle of 60°; (**b**) bending angle of 90°; (**c**) bending angle of 120°; and (**d**) bending angle of 150°.

**Figure 21:**
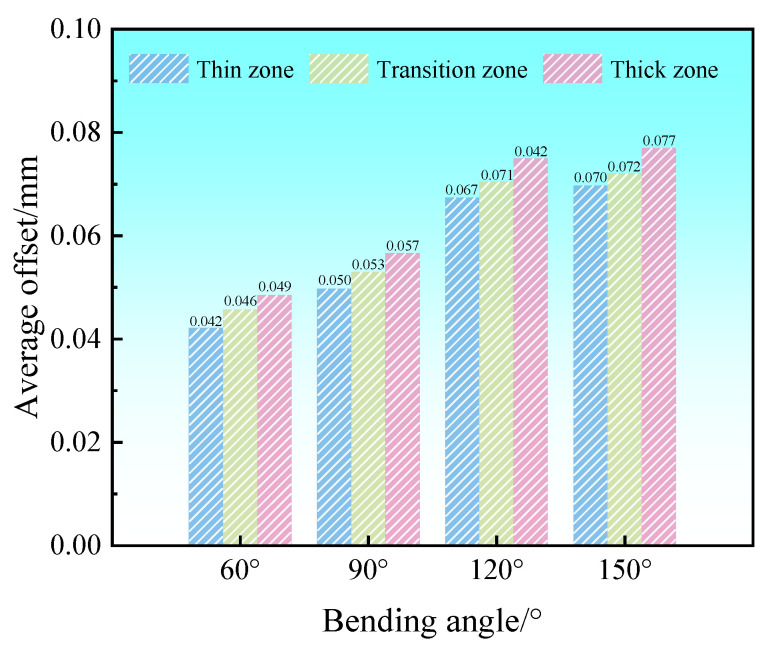
Average offset in each thickness zone of Al-TRB for different bending angles.

In order to better compare the offsets of each thickness zone, the locations of the paths for examining the offsets are set, as shown in Figure 19. Path ①, path ②, and path ③ are the centerlines of the thick zone, transition zone, and thin zone in the width direction, respectively. The offsets of each thickness zone of the Al-TRB during three-point bending are analyzed by studying the offsets of the metal on the three paths in the rolling direction. The results show that the offsets of Al-TRB at the edges of different thickness zones do not differ much, the main gap is in the bending part, and the offsets in the transition zone are the most obvious, as shown in Figure 20. The average offset in each thickness zone after the three-point bending of Al-TRB is shown in Figure 21. It can be seen that there is more metal flow to the thin zone during the three-point bending. As the bending angle increases, the overall offset of the metal increases. The average offset of each zone shows a trend of thick zone > transition zone> thin zone. However, the overall offset during the three-point bending of Al-TRB is not large, and when the bending angle is 150°, the maximum offset is in the thick zone, and its average offset is only 0.077 mm.

#### 4.4.2. Thickness Change in Al-TRB after Three-Point Bending

**Figure 22:**
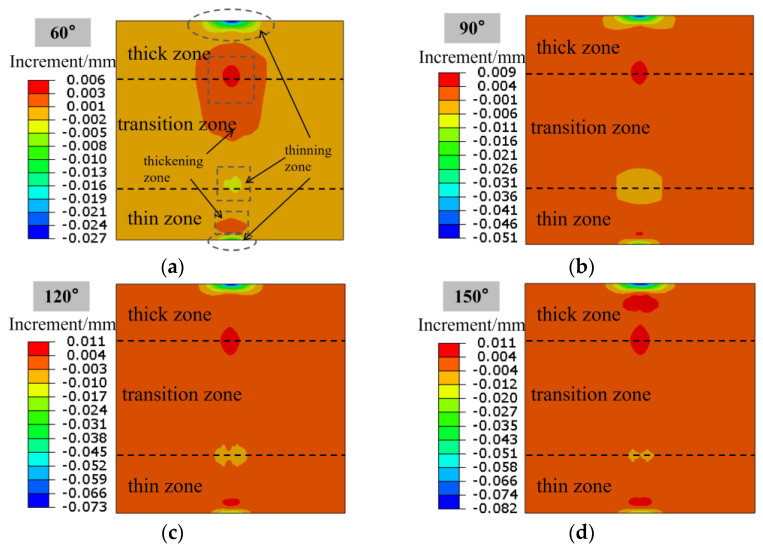
Thickness variation in Al-TRB after three-point bending: (**a**) bending angle of 60°; (**b**) bending angle of 90°; (**c**) bending angle of 120°; and (**d**) bending angle of 150°.

The thickness change in the longitudinal section of the Al-TRB after three-point bending is shown in Figure 22. It can be seen that there is a slight localized thinning and thickening in the bending region of the Al-TRB. The equation for the thinning rate or thickening rate, k, is shown in Equation (1):

k= |t’ − t|/t × 100% (1)

where t’ is the thickness value at each location of the Al-TRB after stamping, and t is the thickness value at the corresponding location of the Al-TRB before stamping; k represents the thinning rate if t’ < t, and k represents the thickening rate if t’ > t.

As the bending angle increases, the thinning and thickening rates increase. When the bending angle is 150°, the maximum thinning rate is 4.08%, and the maximum thickening rate is 0.69%. Overall, during three-point bending, the two rolls only play supporting roles, the Al-TRB bending parts are not made of other fixed conditions, and the metal flow is limited, so the localized thinning and thickening phenomena of the Al-TRB after three-point bending are not obvious.

## 5. Conclusions

In this paper, the strain distribution, spring-back characteristics, and metal flow law of 6000 series Al-TRB during three-point bending were investigated through experiments and finite element simulations. The finite element simulation results are in good agreement with the experimental results. The study results can provide theoretical guidance for designing molds in cold-press forming for the actual production of Al-TRB automotive parts and promote the application of Al-TRB in automotive lightweighting. The major conclusions are as follows:

(1) The prepared Al-TRB has good bending properties, and the deformed region does not crack when bending to 180°. Surface roughening occurs on the outside of the bending region. Since the strain is greater in the thick zone than in the thin zone of the bending process, the surface roughening of the thick zone of the Al-TRB is more obvious than that of the thin zone.
(2) The spring-back angle in the thin zone is higher than that in the thick zone after three-point bending, and the overall spring-back angle of the Al-TRB becomes larger with the increase in the bending degree. When the transition zone of the Al-TRB is centered and the length of the transition zone is certain, as the length of the equal-thickness zone increases, the spring-back angle of the thin zone is larger, while the spring-back angle of the thick zone is smaller. Under the premise of a certain total length of the Al-TRB and the length of the transition zone, the larger the length proportion of the thin zone, the larger the overall spring-back angle of the Al-TRB, and the larger the length proportion of the thick zone, the smaller the overall spring-back angle of the Al-TRB.
(3) There is a slight metal flow phenomenon during three-point bending, which is manifested as the metal in the bending region will flow to the thick zone, and the metal in the edge will flow to the thin zone. As the bending angle increases, the overall offset of the metal increases. The average offset of each zone shows the following trend: thick zone > transition zone > thin zone. At the same time, there are localized thickening and thinning phenomena in the Al-TRB. As the bending angle increases, the thinning and thickening rates increase. When the bending angle is 150°, the maximum thinning rate is 4.08%, and the maximum thickening rate is 0.69%.

## Figures and Tables

**Table 1:** Chemical composition of 6000 series aluminum alloy for experiments (wt.%).

Mn	Cu	Zn	Mg	Si	Ti	Cr	Fe	Al
0.13	0.12	0.01	0.67	0.72	0.02	0.03	0.15	Bal.

**Table 2:** Experimental results of spring-back angles of Al-TRB after three-point bending.

α/°	Thin Zone	Thick Zone
	θ/°	θ/°
60	6.1	4.5
90	7.1	5.3
120	8.2	5.7
150	8.9	6.8

**Table 3:** Four different sizes of Al-TRB.

Four Types	Length/mm			Thickness/mm		Width/mm
	Thin Zone	Transition Zone	Thick Zone	Thin Zone	Thick Zone	
Al-TRB-I	15	30	15	1.0	2.0	60
Al-TRB-II	50	30	50	1.0	2.0	60
Al-TRB-III	25	30	75	1.0	2.0	60
Al-TRB-IV	75	30	25	1.0	2.0	60

**Table 4:** Simulation results of spring-back angles of thin and thick zones at different bending angles.

α/°	Thin Zone			Thick Zone		
	β_1_/°	β_2_/°	θ/°	β_1_/°	β_2_/°	θ/°
60	117.5	123.2	5.7	120.4	124.7	4.3
90	86.7	93.2	6.5	90.5	95.5	5.0
120	58.0	65.9	7.9	62.0	67.2	5.2
150	27.9	36.4	8.5	31.6	38.1	6.5

## Data Availability

Data are contained within the article.
